# Diagnostic Accuracy of Machine Learning AI Architectures in Detection and Classification of Lung Cancer: A Systematic Review

**DOI:** 10.3390/diagnostics13132145

**Published:** 2023-06-22

**Authors:** Alina Cornelia Pacurari, Sanket Bhattarai, Abdullah Muhammad, Claudiu Avram, Alexandru Ovidiu Mederle, Ovidiu Rosca, Felix Bratosin, Iulia Bogdan, Roxana Manuela Fericean, Marius Biris, Flavius Olaru, Catalin Dumitru, Gianina Tapalaga, Adelina Mavrea

**Affiliations:** 1 MedLife HyperClinic, Eroilor de la Tisa Boulevard 28, 300551 Timisoara, Romania; alina.pacurari@medlife.ro; 2 KIST Medical College, Faculty of General Medicine, Imadol Marg, Lalitpur 44700, Nepal; dr.sanketnep@gmail.com; 3 Islamic International Medical College, Faculty of General Medicine, 41 7th Ave, 46000 Islamabad, Pakistan; abdullahmuhammad65@gmail.com; 4 Doctoral School, “Victor Babes” University of Medicine and Pharmacy Timisoara, 300041 Timisoara, Romania; felix.bratosin@umft.ro (F.B.); iulia-georgiana.bogdan@umft.ro (I.B.); manuela.fericean@umft.ro (R.M.F.); 5 Department of Surgery, “Victor Babes” University of Medicine and Pharmacy Timisoara, 300041 Timisoara, Romania; mederle.ovidiu@umft.ro; 6 Department of Infectious Diseases, “Victor Babes” University of Medicine and Pharmacy Timisoara, 300041 Timisoara, Romania; ovidiu.rosca@umft.ro; 7 Department of Obstetrics and Gynecology, “Victor Babes” University of Medicine and Pharmacy Timisoara, Eftimie Murgu Square 2, 300041 Timisoara, Romania; biris.marius@umft.ro (M.B.); olaru.flavius@umft.ro (F.O.); dumitru.catalin@umft.ro (C.D.); 8 Department of Odontotherapy and Endodontics, Faculty of Dental Medicine, “Victor Babes” University of Medicine and Pharmacy Timisoara, Eftimie Murgu Square 2, 300041 Timisoara, Romania; tapalaga.gianina@umft.ro; 9 Department of Internal Medicine I, Cardiology Clinic, “Victor Babes” University of Medicine and Pharmacy Timisoara, Eftimie Murgu Square 2, 300041 Timisoara, Romania; mavrea.adelina@umft.ro

**Keywords:** artificial intelligence, lung cancer, machine learning, diagnostic imaging

## Abstract

The application of artificial intelligence (AI) in diagnostic imaging has gained significant interest in recent years, particularly in lung cancer detection. This systematic review aims to assess the accuracy of machine learning (ML) AI algorithms in lung cancer detection, identify the ML architectures currently in use, and evaluate the clinical relevance of these diagnostic imaging methods. A systematic search of PubMed, Web of Science, Cochrane, and Scopus databases was conducted in February 2023, encompassing the literature published up until December 2022. The review included nine studies, comprising five case–control studies, three retrospective cohort studies, and one prospective cohort study. Various ML architectures were analyzed, including artificial neural network (ANN), entropy degradation method (EDM), probabilistic neural network (PNN), support vector machine (SVM), partially observable Markov decision process (POMDP), and random forest neural network (RFNN). The ML architectures demonstrated promising results in detecting and classifying lung cancer across different lesion types. The sensitivity of the ML algorithms ranged from 0.81 to 0.99, while the specificity varied from 0.46 to 1.00. The accuracy of the ML algorithms ranged from 77.8% to 100%. The AI architectures were successful in differentiating between malignant and benign lesions and detecting small-cell lung cancer (SCLC) and non-small-cell lung cancer (NSCLC). This systematic review highlights the potential of ML AI architectures in the detection and classification of lung cancer, with varying levels of diagnostic accuracy. Further studies are needed to optimize and validate these AI algorithms, as well as to determine their clinical relevance and applicability in routine practice.

## 1. Introduction

Lung cancer accounts for the biggest proportion of mortality resulting from malignancy on the globe [1,2,3]. The majority of patients diagnosed with lung cancer are already in the advanced stages of the disease, which results in a dismal outlook for their future [4,5]. In addition to the advanced stages of diagnosis, the variability of imaging characteristics and histology of lung cancer makes it difficult for doctors to decide which treatment approach will be most effective for both curative and palliative purposes [6].

The imaging characteristics of lung cancer may range from a single microscopic nodule to a ground-glass opacity, several nodules, pleural effusion, lung collapse, and multiple opacities, of which simple and small lesions are exceedingly difficult to detect [7]. Histopathological characteristics include adenocarcinoma, squamous cell carcinoma, small-cell carcinoma, and a wide variety of other less common histological forms by each subgroup [8]. The clinical stage, histology, and genetic aspects of lung cancer all play a significant role in determining the treatment choices available. Nowadays, with the advancement of precision medicine, medical practitioners are required to compile a list of all the patient’s characteristics and gather oncological decision-making teams before making a determination about whether or not to commence chemotherapy, targeted therapy, immunotherapy, and/or any combination of these treatments along with surgery or radiotherapy [9].

In clinical practice, the issue of whether or not the condition should be treated arises on a daily basis. One of the main goals is to identify a model for the detection, categorization, or prediction of lung cancer, although the medical, scientific understanding of the disease is based on the results of clinical tests and the experiences of medical professionals [10]. An important amount of time and energy is consumed for reviewing imaging studies, pathology slides, and reviewing patient documents in order to establish an appropriate diagnosis and identify the most appropriate therapy choices. A reliable prediction and classification model would make the whole process much easier to handle, the role of artificial intelligence (AI) being debatable since the most recent advancement of equipment and software [11].

Artificial intelligence (AI) is a broad term that can be difficult to define, but its applications may involve making predictions or classifications based on previously collected data, such as X-rays, computed tomography (CT), and magnetic resonance imaging (MRI) [12]. The primary components consist of a dataset that is used for training, a pretreatment technique, an algorithm that is used to construct the prediction model, and a pretrained model that is used to expedite the pace at which models are built and inherit past experience [13]. AI built its own logical method to recognize images quickly in order to fulfill its goal of acquiring information swiftly and without any gaps. Computer-aided detection (CAD) systems are neural networks backed by machine learning (ML) algorithms designed to mimic brain-like decisions used in order to ascertain the location of the target site in clinical images. The lesion areas may be marked by AI-based detection techniques, which also helps to eliminate observational oversights [14]. ML algorithms have been proven to facilitate diagnostic medical imaging by differentiating between bronchioles, lung wall, and parenchyma in a clear manner, all while indicating lesions that are abnormal in comparison to the healthy lung zones, helping clinicians to determine alterations with a low threshold for errors [15,16]. Computer-aided diagnostic methods, on the other hand, have given emphasis on identifying nodules as benign or malignant, even for dimensions that go lower than 3 mm in size [17]. 

In the 21st century, artificial intelligence has been more connected to human life, and this tendency can also be seen throughout all fields of medicine. In oncology, particularly for lung cancer, the goal of AI is to provide individualized solutions for each individual patient by taking into account the tumor’s texture, character, stage, and invasion region [18]. Because of the many existing subtypes, lung cancer is the ideal subject for the use of AI. A significant number of studies have indicated the application’s potential use in the identification of lung nodules, as well as diagnostic applications in histology, disease risk stratification, the creation of drugs, and even the prediction of prognosis. Therefore, this systematic review is primarily focused on analyzing and assessing the diagnostic accuracy of existing machine learning AI architectures in the detection and classification of lung cancer, thus providing a comprehensive evaluation of the current state of AI applications in this field.

## 2. Materials and Methods

### 2.1. Review Protocol

This systematic review was conducted in February 2023, utilizing four online databases: PubMed, Web of Science, Cochrane, and Scopus. The review encompassed the literature published up until December 2022. The investigation covered the following medical subject heading (MeSH) [19] keywords: “lung cancer”, “pulmonary nodule”, “pulmonary cancer”, “lung neoplasms”, “thoracic neoplasms”, “AI”, “artificial intelligence”, “machine learning”, “cancer screening”, “neural network”, and “diagnostic imaging”. The search was restricted to English-language journal articles.

The study used a structured and systematic search strategy in compliance with the Preferred Reporting Items for Systematic Reviews and Meta-Analyses (PRISMA) [20] criteria and the International Prospective Register of Systematic Reviews (PROSPERO) [21] guidelines. All pertinent scientific papers examining the accuracy of machine learning AI algorithms in lung cancer detection were incorporated into the analysis. This systematic review was registered on the Open Science Framework (OSF) platform [22].

The primary objective of this systematic review was to address the following research questions:

- What is the accuracy of machine learning AI algorithms in lung cancer detection?
- What machine learning architectures are currently in use?
- What is the clinical relevance of these diagnostic imaging methods?

### 2.2. Data Extraction

The main sources of information for the gathered material included the text, tables, figures, and additional web resources present in the articles. The initial stage of the selection process involved the elimination of duplicate submissions, followed by a thorough examination of each abstract and, ultimately, a complete review of the entire text. Additionally, the reference lists of the collected papers were meticulously inspected to identify relevant content.

In the context of our review, we considered the following variables to be considered for reporting: (1) study characteristics: study number and author, country of the study, the year of study development, study design, and quality assessment; (2) summary of findings: number of patients, AI architecture, the reference group for the ML architecture, and type of lesions identified; (3) performance of the ML architecture: total positive, total negative, false positive, false negative value, and the type of images used for testing; (4) other particularities of the ML architecture: sensitivity, specificity, accuracy, and study particularities. 

We included studies involving adults who were screened for lung cancer incidentally or by screening. The index evaluations included machine learning AI algorithms for analyzing medical images for lung cancer detection. The ML architectures considered for inclusion in the study comprised neural networks and CADs that are built on machine learning models [23,24]. The ML algorithms used radiological parameters to determine the presence of lung cancer and classify the nodules. We excluded the studies employing phantom, histopathology, or microscopic images, non-imaging modalities, and those investigating the accuracy of image segmentation without the augmentation of machine learning architectures. Similarly, studies that assessed other AI algorithms, such as deep learning methods, were excluded in order to allow for a proper standardization of ML algorithms. Other excluded studies were those that assessed other forms of pulmonary disease. Commentaries, editorials, abstract-only assessments, and critiques were also not included in this systematic review. Estimates of diagnostic accuracy, such as true negative (TN), true positive (TP), false negative (FN), and false positive (FP), or sufficient information from which estimates could be computed were required for inclusion.

The diagnostic test accuracy (DTA) measurements comprised sensitivity and specificity, which showed the proportion of individuals with the target condition who had positive test findings and the percentage of those without the disease who had negative test results, respectively. A diagnostic test that was both sensitive and specific was considered to be ideal.

### 2.3. Study Selection and Quality Assessment

**Figure 1:**
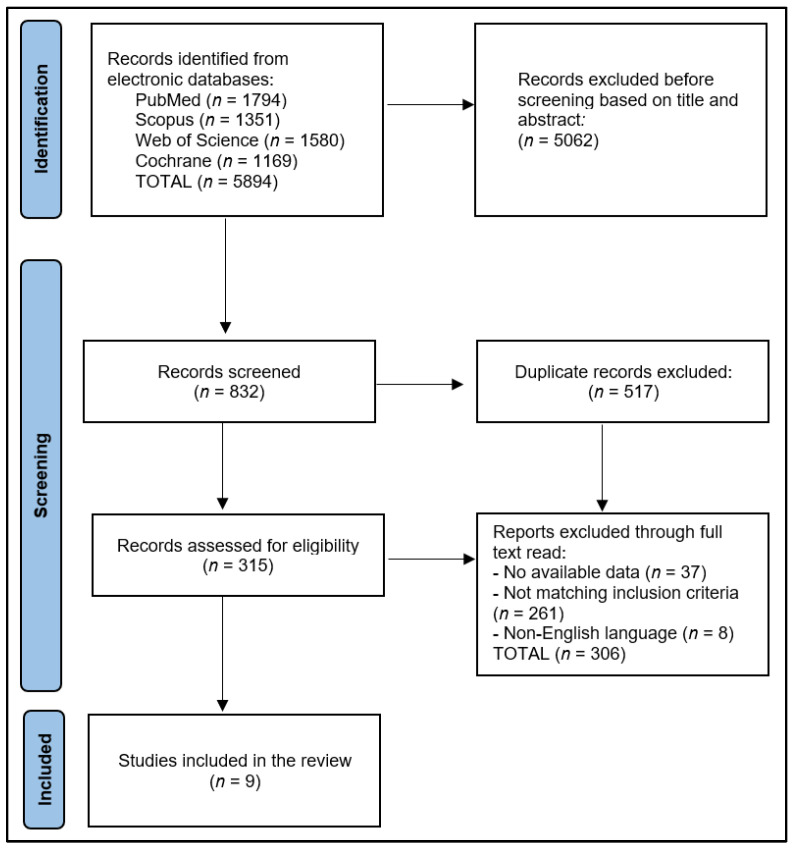
PRISMA flow diagram.

The preliminary search results yielded a total of 5894 articles, out of which 517 were identified as duplicates. After excluding 5062 papers based on their abstracts, 315 full-text articles were assessed for eligibility. Ultimately, nine articles were selected for inclusion in the systematic review, as presented in Figure 1. Based on the Study Quality Assessment Tools provided by the National Heart, Lung, and Blood Institute (NHLBI) [25], two investigators independently evaluated the published material and documented their findings. These tools are tailored to specific study designs, enabling the detection of methodological or design concerns.

For the remaining studies, the Quality Assessment Tool for Observational Cohort and Cross-Sectional Investigations was employed. Each question within the tool received a score of 1 point for “Yes” answers and 0 points for “No” and “Other” responses. Subsequently, the final performance score was calculated. Accordingly, studies with scores ranging from 0 to 4 were considered to be of fair quality, those with scores between 5 and 9 were deemed to be of good quality, and those with a score of 10 or higher were classified as excellent quality. To mitigate inherent biases in the included studies, two researchers were assigned to evaluate the quality of the chosen articles. This approach minimized the risks associated with selection bias, missing data, and measurement bias.

## 3. Results

### 3.1. Overview

Data from nine studies [26,27,28,29,30,31,32,33,34] were analyzed to determine the diagnostic accuracy of machine learning AI architecture in the detection and classification of lung cancer. The studies were conducted in various countries, including Turkey, the United States, Poland, Pakistan, Italy, Bangladesh, and India, and were published between 2014 and 2022. The study designs varied among the selected articles, with five case–control studies [28,29,30,31,33], three retrospective cohort studies [26,27,34], and one prospective cohort study [32]. The quality of the included studies ranged from excellent to fair, with one study deemed excellent [26], three rated as good [29,30,32], and five considered fair [27,28,31,33,34].

A summary of the study characteristics is presented in Table 1. Dandil et al. [26] conducted the earliest study in 2014, which was a retrospective cohort study in Turkey and was the only one rated as excellent in quality. Wu et al. [27] and Kumar et al. [34] also utilized retrospective cohort study designs conducted in the United States and India, respectively, with both being rated as fair in quality. Chauvie et al. [32] carried out a prospective cohort study in Italy, which was rated as good in quality. The remaining five studies were case–control studies conducted in various countries, including Poland [28,31], Pakistan [29], the United States [30], and Bangladesh [33]. The quality of these studies was mixed, with two rated as good [29,30] and three considered fair [28,31,33].

The studies employed various machine learning architectures, including artificial neural network (ANN) [26], entropy degradation method (EDM) [27], probabilistic neural network (PNN) [28,31], support vector machine (SVM) [29,33,34], partially observable Markov decision process (POMDP) [30], and random forest neural network (RFNN) [32]. The type of lesions analyzed in the studies included small-cell lung cancer (SCLC) [26,27], non-small-cell lung cancer (NSCLC) [34], and comparisons of malignant and benign lesions [28,29,30,31,32,33].

The patient population in the studies ranged from as few as 32 patients [34] to as many as 5402 patients [30]. Comparison groups varied among the studies, with some employing microscopic analysis [26,32], expert radiologists’ opinions [29,30,34], random X-rays [28,31], and random slices from healthy lung scans [27,33] as the benchmark for assessing the AI architecture’s performance.

The AI architectures demonstrated promising results in detecting and classifying lung cancer across different lesion types. ANN [26], EDM [27], and SVM [34] showed effectiveness in detecting SCLC and NSCLC, respectively, while PNN [28,31], SVM [29,33], POMDP [30], and RFNN [32] were successful in differentiating between malignant and benign lesions, as described in Table 2. 

### 3.2. Performance Evaluation

The performance analysis of the ML architectures focused on true positives (TP), true negatives (TN), false positives (FP), and false negatives (FN) for each study, as well as the type and number of images used for testing. The studies demonstrated varying degrees of success in the diagnostic accuracy of ML algorithms. Dandil et al. [26] reported a high overall accuracy, with 24 TP, 34 TN, 4 FP, and 2 FN using 128 CT scans. In contrast, Wu et al. [27] reported a slightly higher number of false results, with 30 TP, 26 TN, 10 FP, and 6 FN using 12 high-resolution computed tomography (HRCT) scans, each containing 100–500 slices. Wozniak et al. [28] achieved a balanced performance with 40 TP, 52 TN, 6 FP, and 2 FN using 100 X-rays, of which 80 were from healthy individuals. Khan et al. [29] showed high overall accuracy with 383 TP, 389 TN, 4 FP, and 10 FN using CT scans.

Petousis et al. [30] reported a relatively high number of false positives with 31 TP, 482 TN, 565 FP, and 1 FN using low-dose computed tomography (LDCT) images. Capizzi et al. [31] demonstrated a balanced performance with 43 TP, 68 TN, 7 FP, and 2 FN using X-ray images. Chauvie et al. [32] showed an impressive performance with 18 TP, 1573 TN, 1 FP, and 2 FN using Lung CT Screening Reporting & Data System (RADS) images. Hoque et al. [33] reported a high true positive rate but a low true negative rate with 71 TP, 3 TN, 3 FP, and 1 FN using CT scans. Lastly, Kumar et al. [34] achieved a high true positive rate and low false results with 32 TP, 6 TN, 2 FP, and 2 FN using CT scans, as presented in Table 3.

The findings from Table 4 provide insight into the sensitivity, specificity, accuracy, and particularities of the machine learning architectures used in the nine studies. The sensitivity ranged from 0.81 [34] to 0.99 [29], while the specificity varied from 0.46 [30] to 1.00 [32]. The accuracy of the ML algorithms ranged from 77.8% [27] to 100% [32].

Dandil et al. [26] reported a sensitivity of 0.92, a specificity of 0.89, and 92.3% accuracy. The computer-aided diagnosis (CAD) system they designed involved a combination of self-organizing maps (SOM) and artificial neural networks (ANN). Wu et al. [27] reported lower sensitivity (0.83), specificity (0.72), and accuracy (77.8%) compared to Dandil et al., with their algorithm making 10 false positive predictions and missing 6 cases. Wozniak et al. [28] achieved high sensitivity (0.95), specificity (0.90), and accuracy (92.0%), with their probabilistic neural network (PNN) architecture demonstrating lower computational complexity and the ability to detect low-contrast nodules.

Khan et al. [29] reported impressive results, with a sensitivity of 0.97, specificity of 0.99, and 98.0% accuracy. Their support vector machine (SVM) ML architecture included image contrast enhancement, segmentation, and optimal feature extraction. Petousis et al. [30] achieved high sensitivity (0.97) but relatively low specificity (0.46), and the algorithm was noted to reduce the rate of false positives while maintaining a high rate of true positives. Capizzi et al. [31] reported high sensitivity (0.96), specificity (0.91), and 92.5% accuracy, with their algorithm capable of identifying nodules with a diameter ≤ 20 mm and minimal contrast.

Chauvie et al. [32] achieved a sensitivity of 0.90, a specificity of 1.00, and a remarkable 100% accuracy. Their neural network was the only technique to achieve a high positive predictive value (PPV) without sacrificing sensitivity. Hoque et al. [33] reported a high sensitivity of 0.99 and a specificity of 0.50, with an accuracy of 95.0%. Their improved SVM model effectively identified regions of interest in the lung area where the cancer was localized. Lastly, Kumar et al. [34] reported a sensitivity of 0.81, a specificity of 0.82, and 98.8% accuracy. Their SVM model outperformed other classifiers, such as K-nearest neighbors (KNN), naïve Bayes, and J48, even when using the synthetic minority oversampling technique (SMOTE).

## 4. Discussion

### 4.1. Summary and Contributions

The present study aimed to analyze the diagnostic accuracy of machine learning AI architectures in detecting and classifying lung cancer. Various machine learning AI architectures have the potential to improve the diagnostic accuracy of lung cancer detection and classification. The analyzed studies [26,27,28,29,30,31,32,33,34] demonstrated that AI-based methods could be effective alternatives or supplementary tools to conventional diagnostic approaches, such as microscopic analysis or expert radiologists’ assessments. Moreover, our results, based on data from the nine studies conducted between 2014 and 2022, demonstrated that AI architectures show promise in accurately detecting and classifying lung cancer across different lesion types. These findings are consistent with previous research, which has similarly found AI-based systems to be effective in diagnosing lung cancer [35,36,37]. 

The analysis of the data collected from the nine studies highlighted the potential of machine learning AI architecture for detecting and classifying lung cancer. While the study designs and quality varied, the findings demonstrated a consistent trend toward improved diagnostic accuracy using AI-based methods. Nevertheless, the variations in study design, patient population, AI architecture, and comparison groups highlight the need for further research to establish the most effective AI algorithms and techniques for lung cancer detection and classification. 

Comparing and contrasting the results from the nine studies, it is evident that the ML architectures demonstrated promising results in the detection and classification of lung cancer, with generally high true positive and true negative rates and low false positive and false negative rates. However, the performance varied across studies, with some achieving higher overall accuracy than others. The studies employed various types of imaging, including CT, HRCT, LDCT, X-rays, and RADS, indicating that ML architectures can potentially be effective across a range of imaging modalities. 

In our analysis, the performance of AI architectures varied between studies, with the highest accuracy reported by Chauvie et al. [32] at 100% and the lowest by Wu et al. [27] at 77.8%. These variations may be attributed to differences in study design, quality, AI architecture, and patient populations. A possible explanation for the high accuracy achieved by Chauvie et al. [32] is the use of a random forest neural network (RFNN) in combination with Lung CT Screening Reporting & Data System (RADS) images, which may have improved the detection of malignant and benign lesions.

In comparing our findings with other studies, Narshullah et al. [35] reported an overall accuracy of 94.7% using a deep learning model for lung cancer diagnosis. This is consistent with the high accuracy results reported by Khan et al. [29] and Kumar et al. [34] in our analysis, both of which used support vector machine (SVM) models. Additionally, Ardila et al. [36] found that a deep learning model outperformed expert radiologists in detecting lung cancer, achieving an area under the curve (AUC) of 0.94 compared to 0.88 for human experts. This supports the findings of Petousis et al. [30], who reported a high true positive rate for their AI architecture, despite the relatively low specificity.

The selected studies were conducted in different countries and employed a range of ML architectures, including ANN, EDM, PNN, SVM, POMDP, and RFNN. The findings from these studies were generally promising, demonstrating the potential of AI as a tool for lung cancer diagnosis. Our results are consistent with the growing body of evidence that supports the use of AI for lung cancer detection and classification. For instance, Ardila et al. reported a deep learning algorithm that achieved an area under the curve (AUC) of 94.4% for lung cancer detection on low-dose computed tomography (LDCT) scans [36]. Similarly, a study by Nam et al. showed that a deep-learning-based nodule detection model had a sensitivity of 93.8% and a specificity of 87.4% [37]. These findings indicate that AI architectures have the potential to achieve high diagnostic accuracy in lung cancer detection.

The sensitivity and specificity of the ML architectures in our analysis ranged from 81% [34] to 99% [29] and 46% [30] to 100% [32], respectively. This variation may be attributed to differences in study design, data quality, and the type of ML architecture used. For example, Chauvie et al. [32] achieved a high specificity of 1.00 and an impressive 100% accuracy using the RFNN architecture, while Petousis et al. [30] reported a relatively low specificity of 0.46 using the POMDP architecture. These results suggest that the choice of ML architecture may impact the diagnostic performance of AI systems.

Another study compared the diagnostic performance of two AI methods and found that machine learning was superior to deep learning in early lung cancer detection from medical imaging. The results of deep learning had a sensitivity of 83.7% and a specificity of 82.6%, consistent with previous findings [38]. Deep learning requires large datasets for optimal performance, but some studies used smaller datasets [39,40], reducing statistical power. In cases with insufficient data, traditional machine learning was preferable for accurately detecting lung cancer, although deep learning still held potential for clinical applications with comparable diagnostic accuracy [41].

Deep learning algorithms have been of high interest lately, and various studies attempted to determine their utility as diagnostic tools. In one study [42], the authors compared a deep learning model with an SVM model, which had been widely used in disease prediction, as well as in three of the studies included in our systematic review [29,33,34]. The SVM performed poorly on high-dimensional gene expression datasets, resulting in low prediction accuracy. However, their deep learning model achieved higher accuracy and AUC scores than SVM, as it could automatically learn direct interactions and nonlinear relationships. The results confirmed deep learning’s ability to fit complex relationships without manual intervention, suggesting its increasing importance in disease diagnosis and potential for further development. 

Wang et al. [43] utilized a deep learning model to predict EGFR mutation status in lung adenocarcinoma using CT images. Their model achieved an accuracy of 85.4%. In comparison to these studies that focus on deep learning AI algorithms, their findings also show the potential of deep learning AI in lung cancer detection and classification. However, our findings highlight the superiority of traditional ML when dealing with smaller and insufficient datasets. In such cases, ML architectures may be more suitable for accurately detecting lung cancer in different imaging modalities. While deep learning has demonstrated considerable potential in clinical applications, it requires larger and high-dimensional datasets for optimal diagnostic performance. Therefore, both deep learning and machine learning approaches have their merits and can be complementary depending on the available data and specific use cases.

Our findings also highlight the importance of careful evaluation and validation of AI algorithms for lung cancer diagnosis. In some studies, the ML architectures demonstrated high true positive rates but relatively low true negative rates [33], which may lead to unnecessary follow-up procedures or interventions for patients with benign lesions. Moreover, the studies used various comparison groups, such as microscopic analysis, expert radiologists’ opinions, random X-rays, and random slices from healthy lung scans, which could influence the performance evaluation of the AI systems.

The results of this systematic review not only offer an overview of the current state of machine learning AI architectures used in lung cancer detection, but also provide insights for future research directions. For AI researchers and data scientists, the performance metrics we present here could guide the selection and optimization of models in further studies. For clinicians, understanding the capabilities of these AI tools may open up new possibilities for early lung cancer detection and timely treatment, potentially improving patient outcomes. Moreover, policymakers and healthcare administrators might use this information to inform decisions about incorporating AI diagnostics into routine healthcare, potentially reducing the workload of radiologists and pathologists and improving overall healthcare efficiency.

### 4.2. Study Limitations and Future Directions

Our study has several limitations that should be acknowledged. First, the included studies were heterogeneous in terms of patient populations, imaging techniques, lesion types, and ML architectures used. This heterogeneity may have affected the pooled diagnostic accuracy measures, limiting the generalizability of our findings. Second, the number of studies included in our analysis was relatively small. As a result, our findings should be interpreted with caution, and further research is needed to confirm these results. Moreover, publication bias may have influenced our findings, as studies with positive results are more likely to be published than those with negative results. Additionally, the quality of the included studies varied, with some studies having a relatively small sample size or lacking clear methodological details that may have affected the reliability of our results. Although pooled data analysis can provide more robust and statistically significant insights, the current variability in methodologies, AI architectures, and evaluation metrics among the reviewed studies may limit the applicability and reliability of a pooled analysis. Finally, our study focused on the diagnostic accuracy of AI in detecting and classifying lung cancer but did not explore other important aspects, such as the impact of AI on clinical decision making, patient outcomes, or cost-effectiveness. 

The potential of AI for lung cancer detection and classification is evident; however, further research is needed to optimize ML architectures and evaluate their performance in diverse patient populations. Some future research directions should include the development and validation of AI algorithms in large, multi-center studies that include diverse patient populations to ensure the generalizability of the results. Another important topic is the investigation of the optimal combination of imaging modalities, such as CT, PET, and MRI, and their integration with AI algorithms for improved lung cancer diagnosis. Other possible study hypotheses include the exploration of AI’s role in predicting treatment response, prognosis, and patient outcomes; evaluation of the cost-effectiveness of AI-based lung cancer diagnosis, including the potential reduction in unnecessary follow-up procedures or interventions for patients with benign lesions; and the assessment of the impact of AI on clinical decision making and patient–physician communication, which may lead to better patient-centered care.

## 5. Conclusions

This systematic review has provided a thorough evaluation of the diagnostic accuracy of machine learning AI architectures in lung cancer detection and classification with varying degrees of success, demonstrating their potential and areas for improvement. The study designs and quality varied, while the algorithms employed included ANN, EDM, PNN, SVM, POMDP, and RFNN. The AI architectures were effective in differentiating malignant from benign lesions and identifying small-cell lung cancer and non-small-cell lung cancer. Although the sensitivity, specificity, and accuracy of the AI architectures varied, promising results were demonstrated in many cases, indicating the potential of machine learning algorithms to improve lung cancer detection and classification. However, further research and optimization are needed to enhance the performance and reliability of these AI techniques in real-world settings.

## Figures and Tables

**Table 1:** Study characteristics.

Study and Author	Country	Study Year	Study Design	Study Quality
1 [26] Dandil et al.	Turkey	2014	Retrospective cohort	Excellent
2 [27] Wu et al.	USA	2017	Retrospective cohort	Fair
3 [28] Wozniak et al.	Poland	2018	Case–control	Fair
4 [29] Khan et al.	Pakistan	2019	Case–control	Good
5 [30] Petousis et al.	USA	2019	Case–control	Good
6 [31] Capizzi et al.	Poland	2020	Case–control	Fair
7 [32] Chauvie et al.	Italy	2020	Prospective cohort	Good
8 [33] Hoque et al.	Bangladesh	2020	Case–control	Fair
9 [34] Kumar et al.	India	2022	Retrospective cohort	Fair

**Table 2:** Summary of findings.

Study	Number of Patients	AI Architecture	Comparison Group	Type of Lesions
1 [26] Dandil et al.	47	ANN	Microscopic analysis	SCLC
2 [27] Wu et al.	72	EDM	Random slices from healthy lung scans	SCLC
3 [28] Wozniak et al.	404 for training, 100 for testing	PNN	Random X-rays	Malignant vs. benign
4 [29] Khan et al.	84	SVM	Expert radiologists	Malignant vs. benign
5 [30] Petousis et al.	5402	POMDP	Expert radiologists	Malignant vs. benign
6 [31] Capizzi et al.	320 for training, 120 for testing	PNN	Random X-rays	Malignant vs. benign
7 [32] Chauvie et al.	1594	RFNN	Microscopic analysis	Malignant vs. benign
8 [33] Hoque et al.	78	SVM	Random slices from healthy lung scans	Malignant vs. benign
9 [34] Kumar et al.	32	SVM	Expert radiologists	NSCLC

AI—artificial intelligence; ANN—artificial neural network; NR—not reported; PNN—probabilistic neural network; EDM—entropy degradation method; SCLC—small-cell lung cancer; PNN—probabilistic neural network; SVM—support vector machine; POMDP—partially observable Markov decision process; RFNN—random forest neural network; NSCLC—non-small-cell lung cancer.

**Table 3:** Performance of the ML architecture.

Study	TP	TN	FP	FN	Images Used for Testing
1 [26] Dandil et al.	24	34	4	2	128 CTs
2 [27] Wu et al.	30	26	10	6	12 HRCTs (100–500 slices)
3 [28] Wozniak et al.	40	52	6	2	100 X-rays (80 healthy)
4 [29] Khan et al.	383	389	4	10	CT scans
5 [30] Petousis et al.	31	482	565	1	LDCT
6 [31] Capizzi et al.	43	68	7	2	X-rays
7 [32] Chauvie et al.	18	1573	1	2	RADS
8 [33] Hoque et al.	71	3	3	1	CT scans
9 [34] Kumar et al.	32	6	2	2	CT scans

ML—machine learning; TP—total positive; TN—total negative; FP—false positive; FN—false negative; CT—computed tomography; HRCT—high-resolution computed tomography; LDCT—low-dose computed tomography; RADS—Lung CT Screening Reporting & Data System.

**Table 4:** Other particularities of the machine learning architectures.

Study	Sensitivity	Specificity	Accuracy	Particularities
1 [26] Dandil et al.	0.92	0.89	92.3%	The designed CAD system provides the segmentation of nodules on the lobes with a neural networks model of SOM and ensures classification between benign and malignant nodules with the help of ANN.
2 [27] Wu et al.	0.83	0.72	77.8%	The algorithm makes 10 false positive predictions among 36 tests and misses 6 cases.
3 [28] Wozniak et al.	0.95	0.90	92.0%	This method starts with the localization and extraction of the lung nodules by computing, for each pixel of the original image, the local variance obtaining an output image with the same size as the original image. The PNN architecture has a lower computational complexity, and it can detect low-contrast nodules.
4 [29] Khan et al.	0.97	0.99	98.0%	The ML architecture consists of multiple phases that include image contrast enhancement, segmentation, and optimal feature extraction, followed by the employment of these features for training and testing of SVM.
5 [30] Petousis et al.	0.97	0.46	NR	The ML algorithm reduced the rate of false positives yet preserved a high rate of true positives comparable to that of human experts and identified lung malignancies earlier.
6 [31] Capizzi et al.	0.96	0.91	92.5%	The algorithm can identify nodules with a diameter ≤ 20 mm and minimal contrast.
7 [32] Chauvie et al.	0.90	1.00	100%	Given the various radiological characteristics of nodules on CT and DTS, the lung-RADS category did not improve the diagnostic accuracy of visual examination. The neural network was the only technique to achieve a high PPV without sacrificing sensitivity, as compared with binary visual analysis, logistic regression, and random forest algorithm.
8 [33] Hoque et al.	0.99	0.50	95.0%	The improved SVM model achieved higher accuracy in identifying regions of interest in the lung area where the cancer was localized.
9 [34] Kumar et al.	0.81	0.82	98.8%	The SVM model achieved higher precision than KNN, naïve Bayes, and J48 classifier, with or without SMOTE.

ML—machine learning; CAD—computer-aided diagnosis; SOM—self-organizing maps; ANN—artificial neural network; SVM—support vector machine; NR—not reported; CT—computed tomography; DTS—digital tomosynthesis; RADS—Lung CT Screening Reporting & Data System; PPV—positive predictive value; KNN—K-nearest neighbors; SMOTE—synthetic minority oversampling technique.

## Data Availability

Not applicable.
